# Pemphigus herpetiformis: a single-center tertiary care experience

**DOI:** 10.55730/1300-0144.6020

**Published:** 2024-12-25

**Authors:** Merve Hatun ERKAYMAN, Handan BİLEN, Zeynep KARACA URAL, Elif DEMİRCİ

**Affiliations:** 1 Department of Dermatology and Venereology, Atatürk University Faculty of Medicine, Erzurum, Turkiye; 2 Department of Pathology, Atatürk University Faculty of Medicine, Erzurum, Turkiye

**Keywords:** Pemphigus, immunofluorescence microscopy, basement membrane zone

## Abstract

**Background/aim:**

Pemphigus herpetiformis (PH) is a rare variant of pemphigus with a complex pathogenesis that is often difficult to diagnose. The literature generally presents data based on small case series. This study aims to broaden the understanding of PH by presenting our cases’ clinical, histopathological, and immunological characteristics.

**Materials and methods:**

A retrospective analysis of the medical charts of patients diagnosed with PH between 2008 and 2023 from the Dermatology Clinic at Atatürk University’s Faculty of Medicine was performed. The diagnostic criteria proposed by Kasperkiewics et al. were applied. All patients had lesional and perilesional skin biopsies for histopathologic examination and direct immunofluorescence microscopy. Circulating autoantibodies were detected by ELISA.

**Results:**

Four patients with a diagnosis of PH were observed. The ratio of women to men was 3:1, and the average age of the patients was 59.25 years. Pruritus was present in all patients. The oral mucosa and the scalp were affected in one and two cases, respectively. One patient showed concurrent C3 deposits at the basement membrane zone and intercellular IgG deposits on direct immunofluorescence microscopy. Peripheral eosinophilia was found in 50% of the patients. Dapsone monotherapy led to complete remission in only one patient. At least partial remission was achieved in three patients with systemic steroids ± azathioprine. No concomitant malignant disease was detected.

**Conclusion:**

Establishing a diagnosis of PH may be delayed and requires a high level of clinical suspicion. Unusual direct immunofluorescence findings suggest a complex pathogenesis. A long but less severe disease course can be expected, even if dapsone monotherapy does not result in a satisfactory result in every patient.

## 1. Introduction

Pemphigus herpetiformis (PH) is a peculiar variant of pemphigus originally identified by Jablonska et al. [1]. It accounts for approximately 7% of all pemphigus cases [2]. PH is usually characterized by pruritic, erythematous, and vesiculobullous lesions in a cluster resembling the clinical features of dermatitis herpetiformis. PH diagnosis is based on characteristic clinical signs, intraepidermal separation with/without acantholysis on histology, and the detection of intercellular IgG +/− complement 3 (C3) deposits on direct immunofluorescence microscopy (DIF), as seen in pemphigus [3]. PH is distinct from other pemphigus variants due to its relatively mild disease course and frequent sparing of mucosal areas [3,4]. Desmoglein 1 (Dsg1) is considered the primary target antigen, but desmoglein 3 (Dsg3), desmocollins (Dsc), and other antigens have also been implicated in rare cases [4]. Despite similarities in antigen targets with pemphigus vulgaris, it is unclear why PH’s clinical and pathologic features are different. Autoantibody-augmented signaling pathways that trigger proinflammatory cytokines and chemokines, resulting in the epitope spreading phenomenon, are among the proposed mechanisms driving PH [3,5].

Establishing a PH diagnosis remains challenging despite suggested diagnostic criteria. In a recent study, Costa et al. [4] proposed histopathologic findings as an additional criterion to previous criteria proposed by Kasperkiewicks et al. (clinical and immunologic) [3]. However, the diagnostic significance of histopathologic findings is still being debated as they are highly variable and nonspecific [6]. This highlights the importance of thorough assessments in patients with suspected PH. Therapeutically, dapsone and systemic corticosteroids have emerged as standard treatment options, although the response is variable; therefore, alternative approaches, including various immunosuppressive/immunomodulatory agents, are required in certain cases [4,7].

As PH is a rare disease, most reports are limited to small case series, restricting the generalizability of the results. However, these case series also provide detailed clinical, histopathological, and immunological data and thus contribute to a deeper understanding of the disease. In this case series, four patients diagnosed with PH and treated in a tertiary care center over 15 years are presented to emphasize unique immunopathological findings, such as epidermal basement membrane zone (BMZ) C3 deposition, and evaluate the different clinical presentations and treatment responses. These findings not only contribute to the current literature but also emphasize the complexity and variability of PH presentations.

## 2. Materials and methods

### Patient selection

A retrospective analysis of the charts of the patients diagnosed with PH between 2008 and 2023 at a dermatology clinic at a tertiary university hospital located in the eastern part of Türkiye was performed. Patients fulfilling the diagnostic criteria proposed by Kasperkiewics et al. and available detailed clinical data and follow-up were included [3]. Exclusion criteria included insufficient clinical data or alternative confirmed diagnoses. The informed consent of all patients was obtained.

### Diagnostics

Diagnoses were made according to typical clinical features, such as pruritic, erythematous, and vesicular/bullous/papular lesions grouped together, frequently in an annular pattern and immunologic criteria of intercellular deposition of IgG and/or C3 in the epithelium by DIF or detection of circulating autoantibodies. If DIF is unavailable, circulating autoantibodies against Dsg1 and Dsg3 should be demonstrated using indirect immunofluorescence or enzyme-linked immunosorbent assay (ELISA) [3]. All patients had lesional and perilesional skin biopsies for histopathological examination and DIF microscopy. Circulating autoantibodies were detected via commercially available ELISA kits and considered positive at a cut-off index of 20 U/mL or higher according to the manufacturer’s protocol. Cases with atypical findings were reviewed by dermatologists and a pathologist for consistency.

### Treatment

Given the lack of a standardized protocol for PH, patients were treated based on clinical judgment. Treatments included dapsone, systemic corticosteroids, and azathioprine.

### Data analysis

Quantitative data, including patient demographics, symptom frequency, and treatment outcomes, were summarized. Results were expressed as means or medians, ranges, and percentages. Due to the small sample size, treatment efficacy was analyzed qualitatively.

## 3. Results

The Table summarizes the clinical, histopathological, and immunofluorescence findings, as well as the treatment and outcomes of all patients.

### Demographics

The study included four patients diagnosed with PH. The cohort comprised three females and one male, with a mean age of onset at 59.25 years (range: 43–72 years). The median follow-up time was 4 years (0.4–8.1 years). The median diagnostic delay was 25.5 months (1–72 months). Initial misdiagnosis of pemphigus vulgaris occurred in 50% (2/4) of the patients.

### Clinical presentation

**Figure 1:**
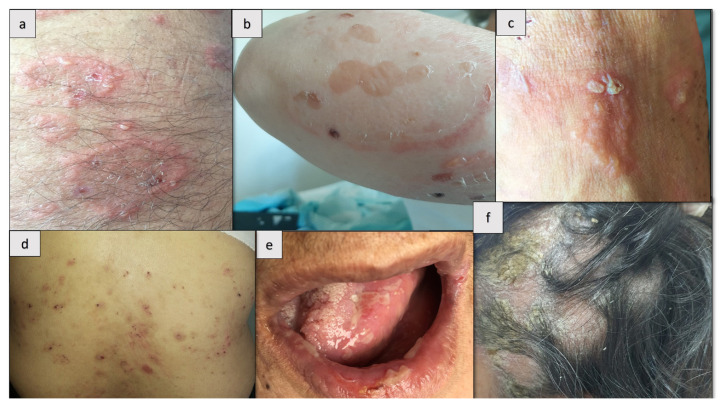
Clinical presentation of patients with pemphigus herpetiformis. Annular erythematous plaques with peripheral vesiculopustules on the abdominal region (a), on the forearm (b), and on the dorsum of the foot (c); erosions and crusts on the back (d), erosions on the tongue and lower lip (e), and desquamated plaques with alopecia on the scalp (f).

All patients (100%) presented with intense pruritus. The blistering lesions were fragile and typically characterized as small vesicular lesions arranged in an annular configuration with erythematous plaques, often affecting the trunk and extremities (Figures 1a–c). However, pustular lesions were also observed in one patient (25%, case 2) (Figure 1a) and bullous lesions in another (25%, case 3) (Figure 1c). In addition to typical lesions on the legs, one patient (25%, case 4) also had excoriated erosions on the trunk (Figure 1d). One patient (25%, case 1) had mucosal involvement, particularly oral erosions (Figure 1e). The scalp was affected in 50% of the cases (Figure 1f).

### Diagnostic and laboratory findings

**Figure 2:**
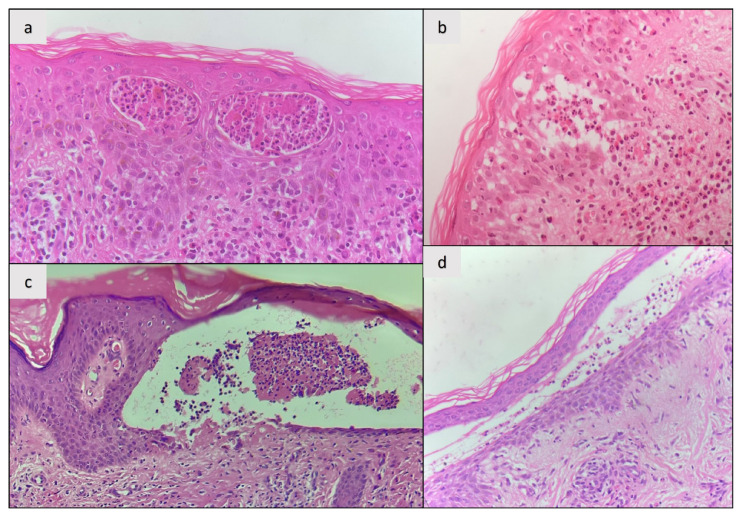
Histopathological findings of patients with pemphigus herpetiformis. (a) Intraepidermal vesicles with neutrophils and eosinophils (hematoxylin and eosin stain; original magnification: ×40); (b) Eosinophil rich spongiotic vesicle in the epidermis (hematoxylin and eosin stain; original magnification ×40); (c and d) Intraepidermal splitting with neutrophils (hematoxylin and eosin stain; original magnification: ×40).

**Figure 3:**
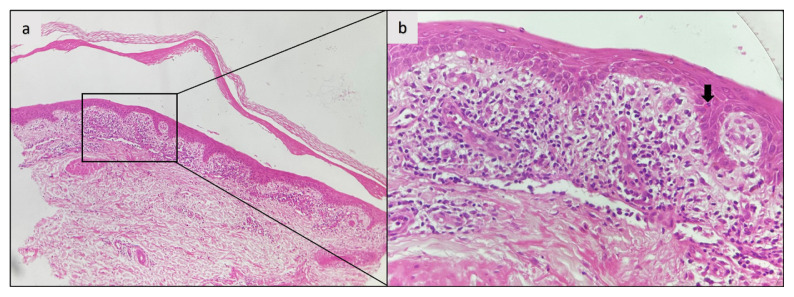
Histopathologic appearance of the first biopsy of case 1 with lichenoid dermatitis. (a) Intraepidermal splitting with some inflammatory infiltrate in the papillary dermis (hematoxylin and eosin stain; original magnification: ×20); (b) Lichenoid dermatitis in the papillary dermis with an epidermal necrotic keratinocyte (arrow)(hematoxylin and eosin stain; original magnification: ×40).

Histopathology: All patients (100%) demonstrated intraepidermal (intraspinous) splitting with no apparent acantholysis. Intraepidermal inflammatory cells contained both eosinophils and neutrophils (Figures 2a and b) in two individuals (50%) and only neutrophils (Figures 2c and d) in two individuals (50%). A lichenoid dermatitis-like infiltration was observed in the initial biopsy of one patient (25%, case 1), who was diagnosed as PV at that time. (Figures 3a and b).

**Figure 4:**
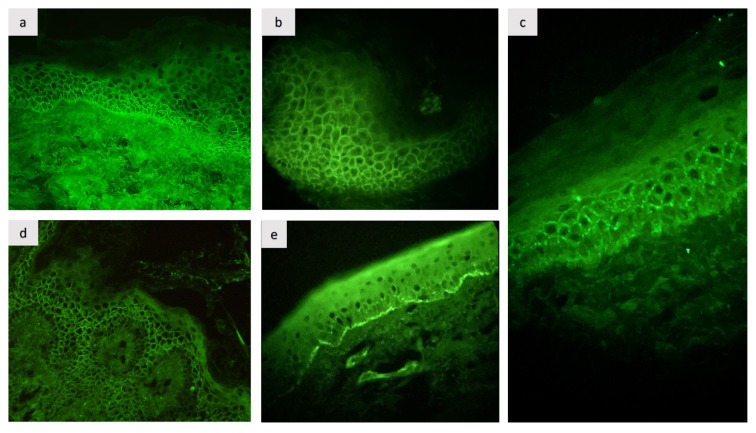
Direct immunofluorescence microscopy shows intercellular IgG deposition in all patients with pemphigus herpetiformis (a, b, c, d) and linear C3 deposition across the membrane in case 1 (e).

DIF: Intercellular IgG deposits in the epithelium were observed in all patients (100%) (Figures 4a–d). In 75% of the patients, additional C3 deposits were noted (3/4). In case 1, two different DIF biopsies were performed. The first biopsy showed intercellular IgG deposits (Figure 4c), while the second showed C3 deposition along the BMZ (Figure 4e).

Serological analysis (ELISA): Anti-Dsg1 autoantibodies were detected in 75% of the patients (3/4), while anti-Dsg3 autoantibodies were observed in 25% (1/4). One patient (25%) tested negative for all tested autoantibodies, including BP180 and BP230. Peripheral eosinophilia was observed in 50% (2/4) of patients.

### Treatment and outcome

Three patients (75%) received dapsone. One (25%) achieved complete remission, one (25%) showed partial remission, and one (25%) was nonresponsive to treatment. One patient (25%) received systemic corticosteroid monotherapy, and complete remission was achieved. Two patients (50%) received a combination of systemic corticosteroids and azathioprine, and partial remission was achieved in both cases.

## 4. Discussion

We describe four new cases of PH and their diagnostic and clinical features. Our case series shows a female predominance in PH, as another recent study shows [4]. However, earlier small case series did not show any sex-specific preference in PH [2,7,8]. The mean age of PH diagnosis was reported as 53 years, comparable to our finding of 59.25 years [4]. Pediatric cases have rarely been reported [9].

PH is a rare, inflammatory type of pemphigus that remains challenging to diagnose due to its atypical features. Kasperkiewics et al. suggested diagnostic criteria mandating the presence of typical grouped itchy vesicular lesions and intercellular IgG and/or C3 deposits on DIF [3]. Recently proposed diagnostic criteria by Costa et al. require the presence of at least one criterion for each clinical sign, histopathological features, and immunological findings to establish the diagnosis [4]. Kasperkiewics et al. did not use histologic findings as a criterion because they are not specifically diagnostic and vary widely; the authors considered DIF the gold standard diagnostic method [6]. PH may often be confused with other types of pemphigus or blistering diseases. Cases 1 and 4 in our study were initially diagnosed as PV. Diagnostic delays in PH are common due to overlapping features with other pemphigus variants [2,4,10]. As histopathological features can be variable and the DIF findings are identical to those of PV, a misdiagnosis is unsurprising, especially if the typical erythematous annular plaques have been overlooked. Distinguishing this less life-threatening form of pemphigus from PV is important to prevent unnecessary overtreatment. However, PH can also occur in the course of PV and pemphigus foliaceous (PF) or vice versa [2,8,11]. Some authors have suggested considering these entities as a continuum that can evolve into both PF and PV [11].

Our patients exhibited the most frequent clinical manifestation of PH: the coexistence of vesiculobullous lesions and erythematous, annular plaques [4]. Although scalp involvement is uncommon in PH, 50% of our patients presented this finding [4]. This highlights the importance of considering PH in patients with atypical presentations. Pruritus was a constant feature in our cases, reported in 86–100% of the patients with PH [4,7]. Mucosal areas are usually spared in PH. In contrast to PV, circulating anti-Dsg3 antibodies do not necessarily require mucosal involvement in PH [12,13]. Nevertheless, in our patients (case 1), the oral mucosa was affected since the initial presentation, and the dominant antibodies were anti-Dsg3 antibodies, which is consistent with the Desmoglein Compensation Theory. In 50% of our patients (2/4), eosinophilia was observed in the peripheral blood, which was previously described at a rate of 15–40% [4,7].

Histopathological findings of PH vary, including intraepidermal splitting with or without acantholysis and concomitant inflammatory infiltration with eosinophils, neutrophils, or both [3,4]. The inflammatory infiltrate, in most cases, is primarily composed of eosinophils. However, in 9% of patients, such as in one of our patients (case 3), only neutrophil granulocytes may be present [4]. Furthermore, a patient’s histologic characteristics may change over time. Remarkably, the initial biopsy of case 1 revealed lichenoid infiltration, an unexpected finding in PH, which was later replaced by the typical histopathological changes of PH [4]. A case of PH with lichenoid infiltrate was previously reported. The authors attributed lichenoid infiltrate to a possible lichenoid drug reaction [13]. Although less likely, they hypothesized that Desmoglein 3-specific CD4+ T cells induce interphase dermatitis based on data from a mice study [14]. Our patient (case 1) also had anti-Dsg3 antibodies and anti-Dsg1, similar to the case reported in the abovementioned study. In contrast to PV, acantholysis is usually absent or minimal in lesional biopsies of PH, while inflammation (predominantly eosinophilic or neutrophilic) is intense [4]. Consistent with this finding, acantholysis was absent during the histopathological examination of the lesional biopsies of our cases. These findings suggest the presence of an alternative pathogenetic mechanism distinct from PV [4]. Antibody-augmented signaling pathways are thought to activate neutrophils and eosinophils via the IgG Fc component, leading to spongiosis and eventual blister formation rather than actual acantholysis in PH [3,5]. In a recent case study, transmission electron microscopy analysis revealed intracytoplasmic vacuoles compressing the nucleus in several keratinocytes, leading to cell rupture. The authors of that study also performed immunoelectron microscopy analysis showing IgG deposition all over the keratinocyte cell surfaces except the desmosomes. They suggested that the main pathogenic antibodies target nondesmosomal parts of Dsg1 [15].

PH shows similar findings (intercellular IgG +/− C3 deposition) with PV on DIF microscopy. The predominant autoantibody is IgG. However, in some patients, a deposition of C3 may also be present (40%) or even occur alone (2%) [4]. In our series, three out of four patients showed concomitant intercellular IgG and C3 deposition. The variation in C3 deposition seen in DIF microscopy has been attributed to the presence of distinct IgG subclasses [4,16]. The most striking feature of our case series was the DIF finding of case 1, which exhibited linear C3 deposition along the BMZ with concomitant intercellular IgG deposition in two different biopsy specimens. However, there was no histopathological subepidermal detachment. Concomitant intercellular and BMZ involvement in DIF has been reported in a few cases of PH [7,9,17–20]. Due to the absence of subepidermal detachment, except in one case, antibodies against BMZ were considered pathogenetically irrelevant [3]. In one case, the simultaneous occurrence of bullous pemphigoid (BP) and PH was observed [20]. In a previous study, the involvement of BMZ in DIF was attributed to a lupus band due to the presence of antinuclear antibodies (ANA). However, the authors detected circulating autoantibodies for BP180 and BP230 in addition to Dsg1 [18]. Our patient also tested negative for ANA, ruling out the lupus band. The exact significance of BMZ involvement in our patient is still unclear. However, this result could be due to an unknown target antigen of unknown significance in BMZ or could indicate a BP that is likely to emerge, given data from prior studies [3,20]. It has been suggested that the strong inflammatory response in PH may lead to the release of new target antigens, known as the epitope-spreading phenomenon, which could also explain our patient’s results [3]. The transformation of PH into other types of pemphigus [2,8,11] and studies reporting PH with concomitant subepidermal autoimmune bullous disease support this phenomenon [4].

The most common autoantibodies found in the sera of patients with PH are those against Dsg1. Accordingly, the most frequent autoantibodies in our case series were anti-Dsg1. Case 1 had autoantibodies against both Dsg1 and Dsg3 that were only found in 4% of reported cases of PH [4]. Case 2 tested negative for antibodies against Dsg1, Dsg3, BP180, and BP230, and the target antigen could not be found. In addition to Dsg1, antibodies to Dsg3, Dsc, BP180, and BP230 have also been detected in rare cases [4]. However, the pathogenic significance of antibodies against BMZ is unknown.

PH is recognized as a variant of pemphigus with a more favorable outcome [3]. In our patients, at least a partial remission was achieved with dapsone and systemic corticosteroids +/− azathioprine. Dapsone monotherapy led to complete remission in only one of three patients receiving the drug. Despite a previous theory positing that the absence of circulating autoantibodies predicts a good response to dapsone, our patient (case 2) failed to show a favorable response to dapsone but did respond to systemic corticosteroids [2]. Our findings underscore the limited efficacy of dapsone monotherapy, which is reportedly effective in 13% of patients with PH. Systemic steroid ± dapsone yielded effective treatment in 44% of patients, and spontaneous remission was also reported [4]. These observations support the less severe course of PH and underline the need for an individualized treatment approach.

PH can occasionally be associated with other autoimmune blistering diseases (10%) and infrequently with comorbidities such as cancer (6%) [4]. In our case series, comorbidities, such as hypertension and hypothyroidism, were regarded as coincidental and not directly linked to PH. Our series also did not reveal any evidence of cancer. Our study has some limitations. It is retrospective, involved a small sample size, and the investigations were limited in terms of target antigens.

In summary, we presented the diagnostic and clinical features of four new cases of PH, including one with a peculiar DIF finding. This implies the presence of a complex and variable pathogenetic mechanism, so there may be subvariants of PH. Dapsone alone has limited efficacy. Dermatologists should consider the diagnosis of PH, particularly in patients who have both pruritus and vesiculobullous skin lesions. They should also consider the atypical clinical and immunological findings to avoid diagnostic delay. Future research should focus on exploring the importance of atypical DIF findings in understanding the pathogenesis of PH and its relationship to epitope spreading, standardizing treatment protocols, and conducting larger, multicenter studies to validate findings and improve generalizability.

## Figures and Tables

**Table:** Clinical and diagnostic features of patients with pemphigus herpetiformis in the study.

Case number	Age/sex	Follow-up (years)	Time to diagnosis (months)	Comorbidities	Clinical findings	Histopathological findings	DIF (intercellular)	ELISA (U/mL)	Eosinophilia	Treatment	Outcome
**1**	64/female	2.5	48	Hypertension, Hypothyroidism	Annular urticated erythema, overlying vesiculobullous lesions, oral mucosal erosions	1. Intraepidermal blistering with vacuolar degeneration, lymphocytic infiltration, necrotic keratinocytes 2. Intraepidermal nonacantholytic splitting with Eo dominant infiltrate	1. IgG, C3 2. IgG Linear BMZ C3	– Dsg3:390 Dsg1:33.8 BP180NC16A: – BP230: –	+	Systemic CS + AZA	PR
**2**	58/male	5	3	–	Annular urticated erythema, overlying vesiculobullous lesions	Intraepidermal neutrophilic pustules, exocytosis of eosinophils and neutrophils	IgG and C3	Dsg1: – Dsg3: – BP180NC16A: –BP230: –	+	1.Dapsone 2.Systemic CS	1. NR 2. CR
**3**	72/female	0.4	1	Hypertension	Annular urticated erythema, overlying vesiculobullous lesions	Nonacantholytic intraepidermal splitting with neutrophils	IgG	Dsg1: 167 Dsg3: –	–	Dapsone	CR
**4**	49/female	8.1	72	Pericardial effusion	Eroded desquamated plaque on scalp, vesiculobullous lesions and erythematous papules	1.Reported as PV 2. Intraepidermal splitting with neutrophils and eosinophils	1. IgG and C3 2. IgG and C3	– Dsg1: 168 Dsg3: –	–	1. Systemic CS + AZA 2. Dapsone 3. Topical steroids	1. PR 2. PR 3. unknown

Eo: eosinophil; C3: complement 3; Dsg1: desmoglein 1; Dsg3: desmoglein 3; BP180NC16A: 16^th^ noncollagenous domain of 180 kDa protein of bullous pemphigoid; BP230: 230 kDa protein of bullous pemphigoid; PV: pemphigus vulgaris CS: corticosteroid; PR: partial remission, NR: no response, CR: complete remission.

## References

[1] Jablonska S Chorzelski TP Beutner EH Chorzelska J Herpetiform pemphigus, a variable pattern of pemphigus International Journal of Dermatology 1975 14 5 353 359 10.1111/j.1365-4362.1975.tb00125.x 1097347

[2] Maciejowska E Jablonska S Chorzelski T Is Pemphigus Herpetiformis an Entity? International Journal of Dermatology 1987 26 9 571 577 10.1111/j.1365-4362.1987.tb02308.x 3327840

[3] Kasperkiewicz M Kowalewski C Jabłońska S Pemphigus herpetiformis: From first description until now Journal of American Academy of Dermatology 2014 70 4 780 787 10.1016/j.jaad.2013.11.043 24472428

[4] Costa LMC Cappel MA Keeling JH Clinical, pathologic, and immunologic features of pemphigus herpetiformis: a literature review and proposed diagnostic criteria International Journal of Dermatology 2019 58 9 997 1007 10.1111/ijd.14395 30900757

[5] Chan LS Vanderlugt CJ Hashimoto T Nishikawa T Zone JJ Epitope spreading: lessons from autoimmune skin diseases Journal of Investigative Dermatology 1998 110 2 103 109 10.1046/j.1523-1747.1998.00107.x 9457902

[6] Kasperkiewicz M Diagnostic criteria for pemphigus herpetiformis International Journal of Dermatololgy 2019 58 11 e216 e217 10.1111/ijd.14584 31286495

[7] Laws PM Heelan K Al-Mohammedi F Walsh S Shear NH Pemphigus herpetiformis: A case series and review of the literature International Journal of Dermatology 2015 54 9 1014 1022 10.1111/ijd.12582 25600350

[8] Santi CG Maruta CW Aoki V Sotto MN Rivitti EA Pemphigus herpetiformis is a rare clinical expression of nonendemic pemphigus foliaceus, fogo selvagem, and pemphigus vulgaris. Cooperative Group on Fogo Selvagem Research Journal of American Academy of Dermatology 1996 34 1 40 46 10.1016/s0190-9622(96)90832-4 8543693

[9] Moutran R Maatouk I Stephan F Halaby E Abadjian G Pemphigus herpetiformis of age of onset at 6 years Dermatology Online Journal 2011 17 6 10 21696690

[10] Ingber A Feuerman EJ Pemphigus with characteristics of dermatitis herpetiformis. A long-term follow-up of five patients International Journal of Dermatology 1986 25 9 575 579 10.1111/j.1365-4362.1986.tb04699.x 3539834

[11] Kühn J Giner T Benoit S Goebeler M Wobser M Pemphigus foliaceus transforming into pemphigus herpetiformis Journal der Deutschen Dermatologischen Gesellschaft = Journal of the German Society of Dermatology 2023 21 6 655 657 10.1111/ddg.15037 37125493

[12] Lebeau S Müller R Masouyé I Hertl M Borradori L Pemphigus herpetiformis: analysis of the autoantibody profile during the disease course with changes in the clinical phenotype Clinical and Experimental Dermatology 2010 35 4 366 372 10.1111/j.1365-2230.2009.03525.x 19874319

[13] Van Rhijn BD Van Der Schaft J Horvath B Van Dijk MR Pas HH Dermpath and Clinic: Pemphigus herpetiformis with vacuolar interface dermatitis and autoantibodies against desmoglein 1 and 3 European Journal of Dermatology 2021 31 3 433 435 10.1684/ejd.2021.4075 34309538

[14] Takahashi H Kouno M Nagao K Wada N Hata T Desmoglein 3-specific CD4+ T cells induce pemphigus vulgaris and interface dermatitis in mice The Journal of Clinical Investigation 2011 121 9 3677 3688 10.1172/jci57379 21821914 PMC3163963

[15] Ishiura N Tamura-Nakano M Okochi H Tateishi C Maki M Herpetiform pemphigus with characteristic transmission electron microscopic findings of various-sized ballooning vacuoles in keratinocytes without acantholysis British Journal of Dermatology 2019 180 1 187 192 10.1111/bjd.16554 29573413

[16] Jerbi A Hachicha H Feki S Bahloul E Sellami K Pemphigus herpetiformis in South Tunisia: a clinical expression of pemphigus foliaceus? International Journal of Dermatology 2018 57 9 1094 1101 10.1111/ijd.14139 30011065

[17] Kavita R Sheenam A Neelima B Jitender BS Unusual direct immunofluorescence (DIF) pattern in pemphigus herpetiformis- A case report Indian Journal of Pathology and Microbiology 2023 66 4 852 854 10.4103/ijpm.ijpm_879_20 38084547

[18] Shimizu K Hashimoto T Wang N Watanabe K Ohata Y A case of herpetiform pemphigus associated with autoimmune hemolytic anemia: detection of autoantibodies against multiple epidermal antigens Dermatology 1996 192 2 179 182 10.1159/000246354 8829509

[19] Palleschi GM Giomi B Herpetiformis pemphigus and lung carcinoma: A case of paraneoplastic pemphigus Acta Dermato-Venereologica 2002 82 4 304 305 10.1080/000155502320323333 12361141

[20] Ohata C Higashi Y Yamagami J Koga H Ishii N Coexistence of pemphigus herpetiformis with IgG antibodies to desmocollin 1 and pemphigoid with IgG antibodies to BP180 C-terminal domain and laminin γ2 JAMA Dermatology 2013 149 4 502 504 10.1001/jamadermatol.2013.1916 23715433

